# Biological activity and dimerization state of modified phytochrome A proteins

**DOI:** 10.1371/journal.pone.0186468

**Published:** 2017-10-19

**Authors:** Peng Liu, Robert A. Sharrock

**Affiliations:** Department of Plant Sciences and Plant Pathology, Montana State University, Bozeman, Montana, United States of America; Instituto de Biologia Molecular y Celular de Plantas, SPAIN

## Abstract

To assess potential physical interactions of type I phyA with the type II phyB-phyE phytochromes *in vivo*, transgenes expressing fusion gene forms of phyA were introduced into the Arabidopsis *phyA* mutant background. When a single c-Myc (myc) epitope is added to either the N- or C-terminus of phyA, the constructs completely complement *phyA* mutant phenotypes. However, addition of larger tags, such as six consecutive myc epitopes or the yellow fluorescent protein sequence, result in fusion proteins that show reduced activity. All the tagged phyA proteins migrate as dimers on native gels and co-immunoprecipitation reveals no binding interaction of phyA to any of the type II phys in the dark or under continuous far-red light. Dimers of the phyA 1–615 amino acid N-terminal photosensory domain (NphyA), generated *in vivo* with a yeast GAL4 dimerization domain and attached to a constitutive nuclear localization sequence, are expressed at a low level and, although they cause a *cop* phenotype in darkness and mediate a very low fluence response to pulses of FR, have no activity under continuous FR. It is concluded that type I phyA in its Pr form is present in plants predominantly or exclusively as a homodimer and does not stably interact with type II phys in a dimer-to-dimer manner. In addition, its activity in mediating response to continuous FR is sensitive to modification of its N- or C-terminus.

## Introduction

The presence of light triggers developmental programs in plants that result in gene expression and growth patterns adapted for harvesting solar energy, competing with neighboring vegetation, and engaging in photoautotrophic metabolism. These light-induced changes in morphology, physiology, and metabolism are referred to as photomorphogenesis. The environmental light signals that trigger photomorphogenesis are sensed by discrete classes of photoreceptor molecules, which are responsive to UV, blue, red, and far-red wavelengths [1–4]. Phytochromes (phy) are dimeric chromoproteins that function as red(R)/far-red(FR) photoreceptors in plants, algae, and bacteria [5–8]. The bilin-linked phy chromoproteins photoconvert between an inactive R-absorbing Pr conformation and an active FR-absorbing Pfr conformation [9, 10]. In some cases, phy-related receptors from cyanobacteria, fungi, and algae are activated by wavelengths from a broader range of colors [11, 12], but in angiosperm plants phys are classically R/FR-photoconvertible.

Plant phys show light-dependent cellular re-localization between the cytosol and the nucleus [2, 13]. Photons of R photoconvert Pr to Pfr, which is translocated to the nucleus and initiates signaling, whereas photons of FR photoconvert Pfr to Pr and terminate signaling. The apoprotein moieties of plant phys are encoded by a small family of genes, of which the five genes designated *PHYA*-*PHYE* in Arabidopsis are representative [14]. Each of the 1100–1200 amino acid phyA-phyE polypeptides covalently binds a linear tetrapyrrole chromophore called phytochromobilin, enfolding it in a globular protein structure consisting of the approximately 600 N-terminal amino acids, which is referred to as the photosensory domain (PSD). The C-terminal halves of plant phy proteins contain conserved domains and are known to mediate aspects of signaling function and nuclear localization [5, 10]. Details of phy signal transduction mechanisms have been elucidated, many centering on phy-mediated release from the repression of photomorphogenesis carried out by nuclear proteins including the COP/DET/FUS complex and the Phytochrome Interacting Factor (PIF) transcription regulators [8, 15].

Plant responses to R and FR can be categorized by the wavelength, irradiance, and duration requirements for their induction, the degree to which that induction can be reversed by exposure to FR, and the kinetics of those interactions [16]. Among these, low fluence R/FR-reversible responses (LFR) can be triggered by a pulse, or a series of pulses, of R (1–1000 μmol m^-2^ s^-1^) and, importantly, this activation can be cancelled with a subsequent pulse, or a series of subsequent pulses, of FR. In Arabidopsis, the apoproteins for the phys that mediate R/FR-reversible responses, and responses to the R:FR light ratio called the shade-avoidance responses, are encoded by the *PHYB*-*PHYE* genes and are designated type II phys [1, 17]. In contrast to this, there are plant responses that are induced only by long-duration continuous irradiation with photons that can be from a relatively broad spectrum of wavelengths of visible light, including blue (B), R, or FR photons. These responses are not FR-reversible and are called high irradiance responses (HIR). There are also responses that are induced by production of very small amounts of Pfr in the plant, by exposure to low levels of either R or FR light, called very low fluence responses (VLFR). The FR-HIR and VLFR are mediated by type I phyA [3, 18]. Type I phyA is markedly light-labile, with its Pfr form being ubiquitylated and degraded relatively rapidly by the proteasome [19] while the type II phyB-E forms are much more stable as Pfr [20].

Many light responses seen at the whole plant developmental or physiological levels have components of both type I and type II phy regulation. Moreover, among the downstream phy signaling effector proteins that have been identified, such as members of the PIF family of transcription factors and components of the COP1/SPA protein degradation machinery, overlapping sets of downstream proteins are involved in type II R/FR-reversible and type I FR-inducible responses [15, 21–23]. Therefore, the diversity of dimeric phytochrome structures present in plant cells and the extents to which different phys function together, separately, or antagonistically are important to developing an integrated understanding of R/FR light response. This has been addressed genetically through the construction of multiply mutant phy-deficient lines and it is evident that there is both simple overlap and various levels of synergism/interaction in the functions of the phys [24, 25]. This raises the question of whether phys physically associate with each other. Phys require dimerization to be active and forcing dimerization of the phyA or phyB N-terminal PSDs, by fusing them to dimerization domains from unrelated proteins, is sufficient to produce biologically active phy chimeric molecules [26, 27]. This indicates that much, though not all, of the signaling function of phys resides in this N-terminal PSD region and is active only when two regions are dimerized. The Arabidopsis type II phyB-phyE receptors form both homodimers and heterodimers in plants, giving rise to a diverse collection of different receptor forms [28, 29], each of which has the possibility of having evolved unique functions. Using heterologous homo- and heterodimerization protein domains, it has been shown that various dimer combinations of the phyB-phyE N-terminal PSDs are active in regulating plant R light responses [30, 31]. One major question that remains is whether type I phyA can dimerize with the type II phys or, alternatively, whether phyA dimers physically associate with type II dimers. We address these questions using immunoprecipitation with fully-active phyA apoprotein constructs and find that, in its Pr form, phyA is detectable only as a homodimer and does not show apparent interaction with the type II phys.

## Materials and methods

### Plant material and growth conditions

All experiments were performed in the Arabidopsis Landsberg *erecta* (L*er*) genetic background, using the *phyA*-201 mutant allele. For seedling growth experiments, seeds were surface sterilized and plated on Murashige and Skoog medium containing 1.2% agar without sucrose. For most experiments, the plates were stratified in the dark for 3–5 days at 4°C, exposed to fluorescent light at room temperature for 3 h to induce uniform germination, returned to the dark at room temperature for one day, and then transferred to the growth conditions described in the figure legends. Hypocotyl lengths were determined by laying out 20–30 5-day-old seedlings per light treatment on agar plates, photographing them, and measuring the hypocotyls using Image J version 1.37 software (National Institutes of Health). FR high irradiance (FR-HIR) experiments were performed using FR (735 nm) light supplied by LEDs in an E-30LED growth chamber (Percival, Perry, IA) at 22°C. For VLFR experiments, stratified seeds were given a saturating pulse of 5 min 30 μmol m^-2^ s^-1^ R (670 nm) to induce germination, incubated in darkness at 22°C for 24 h, then exposed to hourly pulses of FR (3min, 31 μmol m^-2^ s^-1^) for 3 days at 22°C and hypocotyl lengths were measured. For analysis of the *constitutive photomorphogenesis* (*cop*) phenotype, seeds were stratified for 3 days, germination was induced with a 5 min pulse of 30 μmol m^-2^ s^-1^ R, and seedlings were grown for 4 days in darkness. In some experiments as described in Results, the high level (~70%) of phy Pfr produced by the R pulse used to induce germination was reduced to a low level (~1%) by 3 h of darkness followed by 5 min of FR prior to the 4 days of dark growth. Days to flowering were determined for plants grown in a Conviron chamber at 22°C under short days with a low fluence FR-enriched day extension consisting of 8 h 200 μmol m^-2^ s^-1^ (400–700 nm) fluorescent light, 8 h 2 μmol m^-2^ s^-1^ (400–700 nm) incandescent light, and 8 h darkness [32]. Flowering date was defined as the day of first appearance of the inflorescence apex from at least 10 plants for each transgenic line.

### Plasmid construction and plant transformation

All plant transformation plasmids were constructed in vectors derived from pBI123 [33] and carry a selectable marker gene conferring kanamycin-resistance. Using the coding sequence fusion junctions shown in S1 Fig, the full-length *PHYA* cDNA sequence from Arabidopsis ecotype Col was translationally fused at either its N-terminus or its C-terminus to the YFP coding sequence or the myc1 or myc6 epitope tags. These coding sequences were placed under the control of either the CaMV 35S promoter or an Arabidopsis ecotype Col *PHYA* promoter fragment consisting of 2.8 kb of genomic sequence upstream of the start codon. The 35S:NphyA-yeast GAL4-myc6-NLS fusion transgene (NphyA-GAL) was constructed from a subclone of the Col ecotype cDNA sequence in the directed-homodimerization vector described previously [31]. Arabidopsis plant transformations were performed by the floral dip method using *Agrobacterium tumefaciens* strain GV3101. Multiple independent single-locus-insert lines were isolated for each construct and experiments were performed with homozygous T3 or T4 progeny generations.

### Protein extraction, denaturing gel and immunoblot analysis, native gel electrophoresis, and immunoprecipitation

Seedling protein extracts were prepared by grinding 1–3 gms of fresh or frozen seedings in non-denaturing extraction buffer (25 mM Tris (pH 7.5), 10 mM NaCl, 5 mM EDTA, protease inhibitor cocktail—Roche Diagnostics) for 30 seconds in a mortar on ice with 50 μl of a water suspension of 100–150 μm acid-washed glass beads. The buffer volume-to-tissue weight ratios were 1 ml:gm for dark-grown tissue and 2 mls:gm for light-grown tissue. The lysates were centrifuged at 12,000 rpm for 5 min at 4°C and the supernatant was removed and used directly for experiments. Protein content of the extracts was determined using the Bradford protein assay (Bio-Rad). For denaturing gel analysis, aliquots of the extracts were mixed with an equal volume of 2X SDS sample buffer, heated at 95°C for 5 min, and immediately loaded on SDS-PAGE gels or the extracts were frozen at -80°C for later use. Samples of 75–100 μg of seedling protein, depending upon the antibody used to detect the target protein, were separated on 6–7% SDS-PAGE gels. Immunoblotting with the anti-myc 9E10, anti-phyA 073d, or anti-phyD 2C1 monoclonal antibodies was carried out as described [34]. Antibodies were detected with SuperSignal West Pico reagents (ThermoFisher Scientific). For native gel electrophoresis, dark-grown seedling extracts were prepared in non-denaturing extraction buffer as described above and the proteins were separated on 4–20% gradient PAGE gels in Tris/borate/EDTA buffer for 40 h at 4°C. Gels were blotted and probed with the anti-phyA 073d antibody. For immunoprecipitation (IP) analysis, extracts were prepared by grinding 1–3 gms of fresh seedling tissue in an ice-cold mortar in IP buffer (50 mM Tris (pH 8.0), 150 mM NaCl, 0.1% IGEPAL CA-630, protease inhibitor cocktail). The lysates were centrifuged at 12,000 rpm for 5 min at 4°C and the extract supernatant was removed. 50 μl of tissue culture supernatant of the anti-myc monoclonal line 9E10 (gift of Seth Pincus, Louisiana State University) were added to 1 ml of seedling extract and the mixture was incubated for 60 min on ice with gentle mixing. 40 μl of protein A-agarose beads (Santa Cruz Biotechnology) were added, the mixture was incubated on ice with occasional mixing for 1 h, and the beads were pelleted by centrifugation at 5000g for 30 sec at 4°C. The beads were washed four times in 500 μl of extraction buffer. Proteins bound to the beads were eluted by heating at 95°C for 5 min in 2X SDS sample buffer and the beads were pelleted. The eluted proteins were analyzed by fractionation on 6% SDS/PAGE, blotting to nitrocellulose, and probing with the anti-phyA 073d, anti-phyB B6B3, anti-phyC C11 and C13, anti-phyD 2C1, and anti-phyE 7B3 monoclonal antibodies [35]. For each IP experiment, a set of gel lanes was loaded with the protein extract on the basis of protein concentration and a set of gel lanes was loaded with IP samples as equivalent volumes from precipitations performed in parallel.

## Results

**Fig 1:**
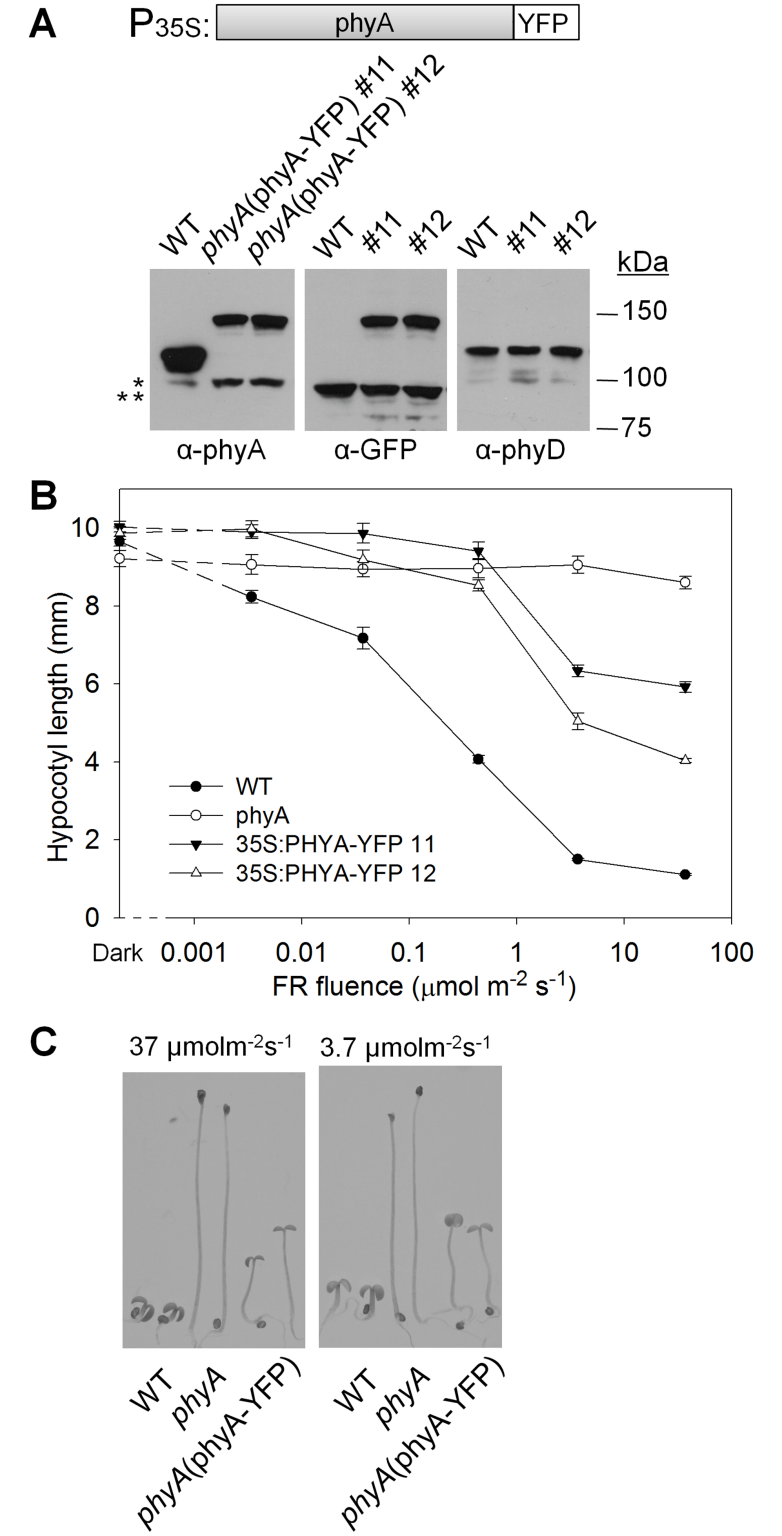
Expression and activity of the 35S:phyA-YFP transgene. (A) Diagram of the 35S:phyA-YFP transgene and immunoblot analysis of the levels of the phyA-YFP protein compared to endogenous phyA in dark-grown seedlings. Protein blots were probed with antibodies to phyA, GFP, and phyD. The anti-phyA antibody detects a degradation product (*), which is more abundant in the phyA-YFP lines than in WT, and the anti-GFP antibody detects a cross-reacting protein (**). The anti-phyD blot is a loading control. (B) Fluence response curve of the activity of phyA-YFP in the FR-HIR. Seedlings of the indicated genotypes were grown for one day in the dark and 4 days at 22°C under continuous FR light of the indicated fluence and hypocotyls were measured (means ±SE; n = 20–30). In unpaired t-test analysis, all *p*-values are greater than 0.05 for dark-grown seedlings. All *p*-values are greater than 0.005 when comparing the *phyA* mutant to the 35S-phyA-YFP lines when they are grown under 0.44 μmol m^-1^ s^-2^ FR or lower fluence. All p-values are lower than 1 X 10^−5^ in other comparisons except WT vs. *phyA* at 0.0034 μmol m^-1^ s^-2^ FR, where the p-value is 0.009. (C) Photographs of representative seedlings are shown to illustrate cotyledon size and opening.

**Fig 2:**
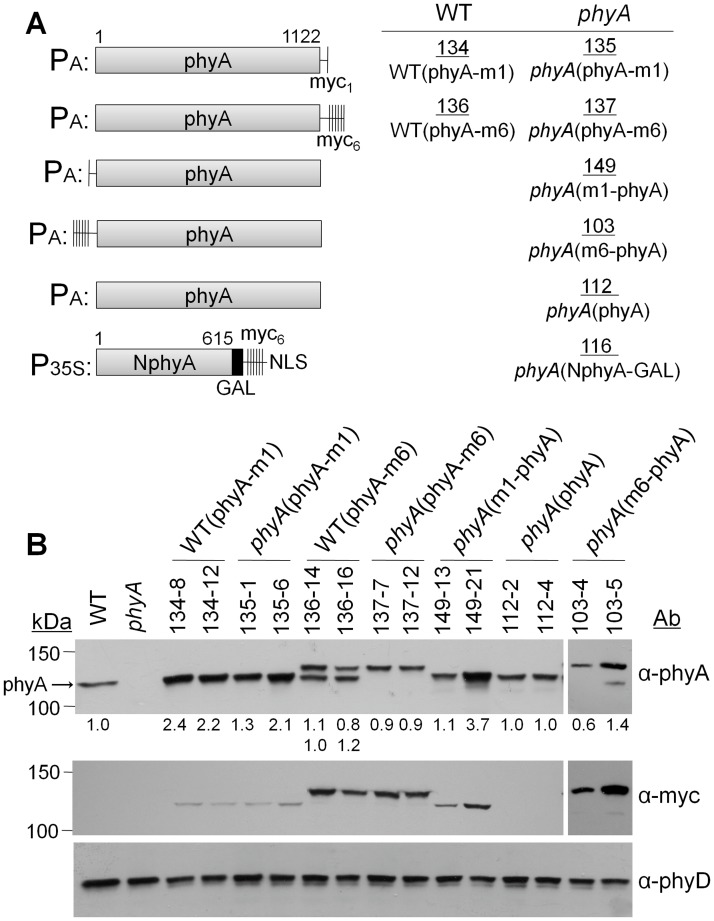
Structures and expression levels of epitope-tagged phyA fusion genes. (A) Diagrams of the transgenes used in these studies and their inclusion in different transgenic lines are illustrated. (B) Immunoblot analysis of the levels of the transgene-encoded proteins compared to native phyA in the WT line in 5-day-old dark-grown seedlings. Protein blots were probed with antibodies to phyA, the c-Myc epitope, and phyD. The numbers below the anti-phyA blot lanes indicate the expression levels of transgene-encoded proteins in the transgenic lines relative to the normal wild-type phyA level, as determined by densitometry analysis in which densitometry values for the bands on the anti-phyA immunoblot were normalized to densitometry readings for bands on the anti-phyD control blot for each lane. The values for the 134 WT(phyA-m1) lines include both the native phyA present in the line and the phyA-myc1 transgene product, since they migrate at the same position in the gel.

One approach to testing for potential heterodimerization of phyA with the type II phyB-E proteins is to construct a fully active epitope-tagged version of phyA, express it in transgenic plants, and use the anti-epitope antibody to perform a co-immunoprecipitation assay. It has previously been observed that the phyA-GFP (green fluorescent protein) or phyA-YFP (yellow fluorescent protein) protein fusions expressed from the CaMV 35S promoter are biologically active in transgenic tobacco and Arabidopsis plants [36–38]. Therefore, a 35S:phyA-YFP gene was introduced into *phyA* null mutant plants. Fig 1A shows that, in homozygous lines, this transgene produces a lower level of protein in the dark than the endogenous *PHYA* gene in the wild-type (WT). Fig 1B shows that, the phyA-YFP protein does not completely complement the *phyA* mutant FR-HIR long-hypocotyl phenotype in our lines, although it has some activity at high FR fluences, and is moderately active in FR induction of cotyledon opening. Why the activity the 35S:phyA-YFP construct used here is lower than that described by others using similar transgenes [38] is not clear but, to test for potential heterodimerization with type II phys, utilization of a completely active target phyA was deemed advantageous. To accomplish this, as shown in Fig 2A, various c-Myc (myc) epitope tagged sequences were added to the ends of phyA and expressed from the native *PHYA* promoter. The expression levels of the transgene products under dark growth conditions relative to endogenous phyA in the WT were determined by immunoblot analysis with anti-phyA antibody (Fig 2B). In most cases, the transgene-encoded proteins were expressed at 0.8-to-2.5 fold that of endogenous phyA. As expected, when blots were probed with an anti-myc antibody, the myc6(m6)-tagged proteins were detected with higher sensitivity than the myc1(m1)-tagged versions (Fig 2B). Expression of the tagged proteins was also similar to that of endogenous phyA when seedlings were grown under continuous FR, although the myc6-phyA protein showed pronounced degradation to a size similar to that of un-tagged phyA under those conditions (S2 Fig).

**Fig 3:**
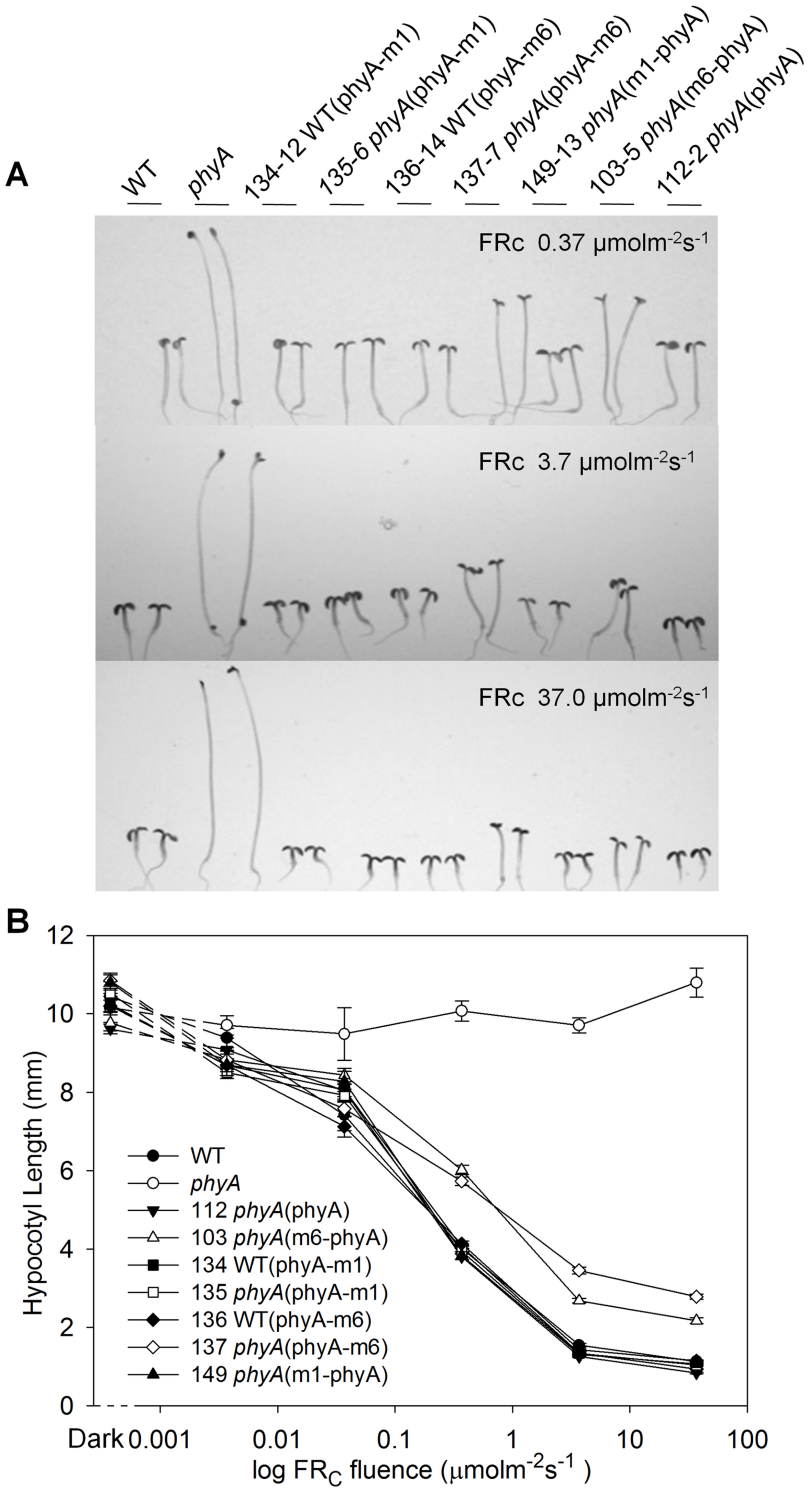
Activities of the epitope-tagged phyA proteins under continuous FR light. (A) Morphologies of seedlings of the indicated genotypes grown for one day in the dark and 4 days at 22°C under three different fluences of continuous FR. (B) Continuous FR fluence response curves of hypocotyl length in transgenic lines expressing modified phyA proteins (means ±SE; n = 20–30).

**Fig 4:**
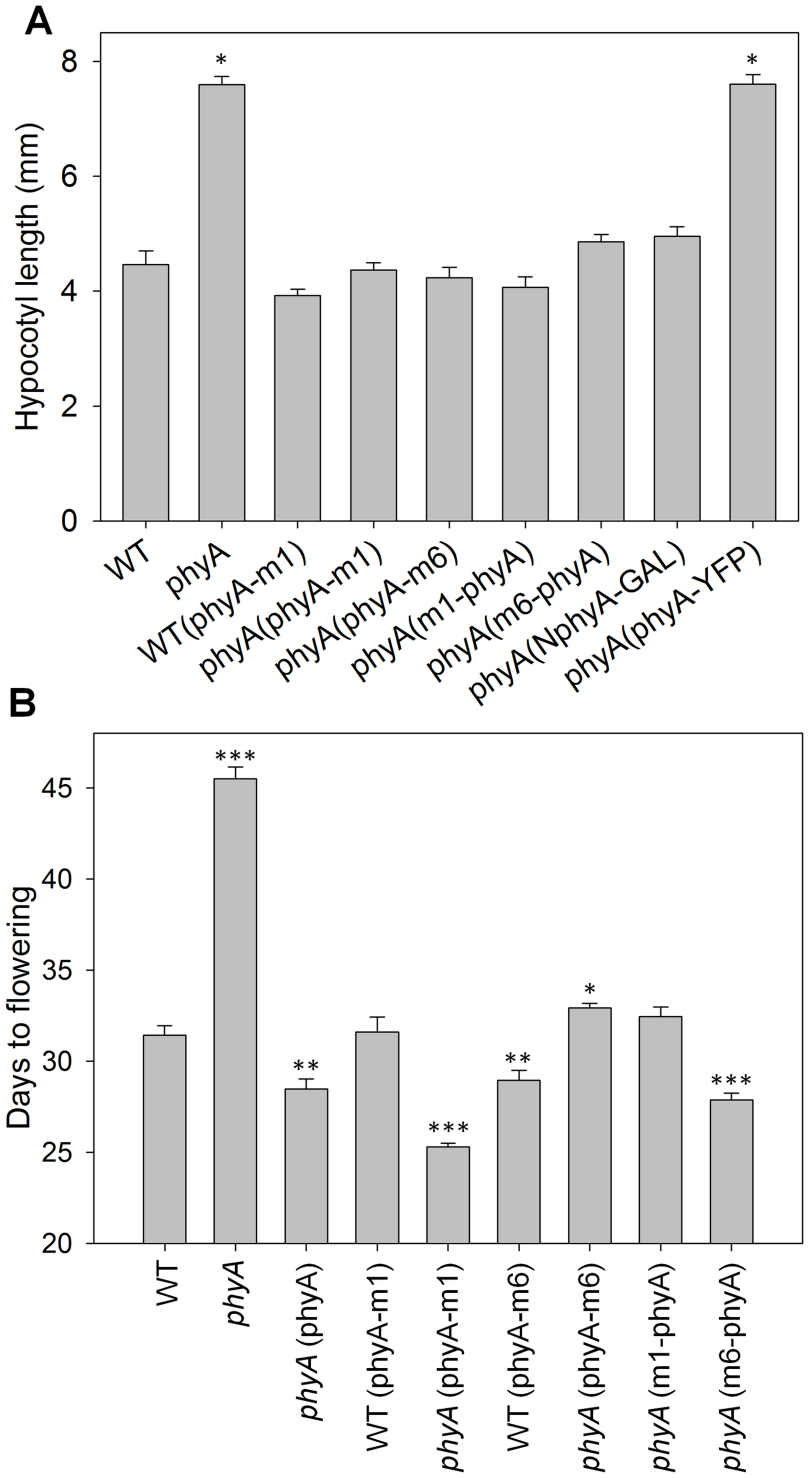
Activities of epitope-tagged phyA proteins in regulating VLFR and extended-day flowering time. (A) Hypocotyl lengths of seedlings grown for 1 day in darkness followed by 3 days at 22°C under FR pulses (3 min 31 μmol m^-2^ s^-1^ FR + 57 min dark) (means ±SE; n = 20–30). Asterisks indicate significant differences (*p value < 0.05) relative to the wild type plants. (B) Days to flowering under short days with low fluence FR-enriched day extension (22°C; 8 h fluorescent light at 200 μmol m^-2^ s^-1^, 8 h incandescent light at 2 μmol m^-2^ s^-1^, 8 h dark) (means ±SE; n = 13–19). Asterisks indicate significant differences (*p value < 0.05, **p < 0.01, ***p < 0.001) relative to the wild type plants.

Fig 3 shows the seedling responses of the transgene-expressing lines to continuous FR. At all FR fluences, lines expressing untagged or myc1-tagged phyA show the same extent of cotyledon opening and inhibition of hypocotyl elongation as the WT line. In contrast, lines expressing myc6-tagged versions of phyA, while clearly sensitive to FR, are deficient in their response to FR at fluences above 0.1 μmol m^-2^ s^-1^. Hence, the large myc6 tag interferes to some extent with phyA signaling through the FR-HIR seedling elongation response. To test whether myc6-tagged phyA interferes with the HIR activity of endogenous phyA, the *PHYA*:phyA-myc6 gene was introduced into the WT background. Fig 3 shows that expression of this protein does not cause a dominant negative effect under FR. To assess the activities of the tagged phyAs in signaling through the VLFR, lines were grown under hourly pulses of FR [39, 40]. Fig 4A shows that all the myc-tagged phyAs mediate a robust VLFR, whereas the 35S:phyA-YFP fusion is not active in VLFR. Flowering time, a mature plant response, was also measured in the myc-tagged phyA lines. Fig 4B shows that, under extended low fluence FR-enriched day conditions (8 h fluorescent/8 h low incandescent/8 h dark) [32], several of the myc-tagged phyA lines flower somewhat earlier than the wild-type and they all clearly show complementation of the *phyA* mutant late flowering phenotype. Therefore, the inhibitory effect of addition of a large epitope myc6 tag to the ends of the phyA apoprotein on its signaling function is seen most strongly in seedling FR-HIR responses. Type I phyA is light labile and transfer from dark to R light results in reduction of the level of the receptor with a half-life of 0.5–2 hr [3]. One possible cause for reduced signaling activity of the myc6-tagged phyA proteins would be if they were more rapidly degraded than WT phyA as Pfr. Supporting S3 Fig shows that these tagged proteins are in fact more stable than the native molecule following transfer to Rc. The native phyA protein in the WT(phyA-m6) lines, which express both native and tagged phyA, is degraded more slowly than in the WT and at a very similar rate to the transgene-encoded phyA-myc6. This may reflect competition between the two phyA proteins for R-induced ubiquitylation.

**Fig 5:**
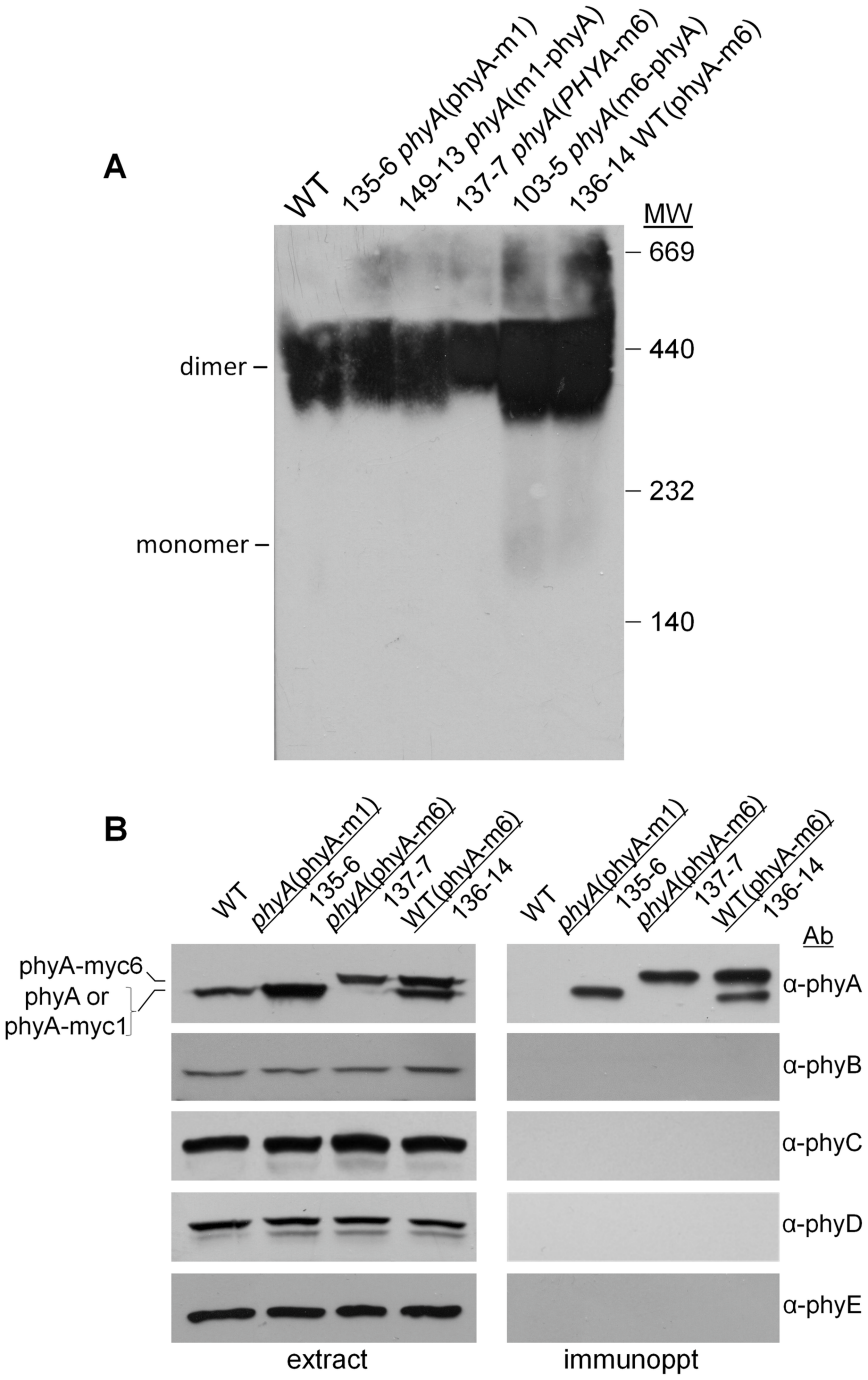
Native gel and co-immunoprecipitation analyses of the dimerization state of epitope-tagged phyA proteins. (A) Native gel analysis of myc1- and myc6-tagged phyA protein levels in transgenic seedlings in the WT or *phyA* backgrounds. Non-denatured extracts of dark-grown seedlings were fractionated on a 4–20% native PAGE gel and a blot of the gel was probed with the anti-phyA antibody. The positions at which monomeric and dimeric phyA migrate on native gels are indicated. (B) Immunoblot analysis of dark-grown seedling extracts and anti-c-Myc antibody immunoprecipitates from WT and lines expressing the indicated transgenes. The WT(phyA-m6) extract contains both native phyA and the higher molecular weight myc6-tagged phyA.

Heterodimerization among the Arabidopsis phyB-phyE phytochromes generates increased structural diversity among the type II phytochromes [28, 29]. The epitope-tagged phyA lines make it possible to assess potential physical interactions of phyA with type II phyB-E by co-immunoprecipitation. Fig 5A shows that the myc-tagged phyA molecules migrate as dimers in a non-denaturing electrophoresis gel. In Fig 5B, immunoprecipitation of the tagged phyA proteins shows that dimers form between phyA-myc6 and endogenous phyA in the WT(phyA-m6) line, as expected. Three different myc-tagged phyA proteins were used to test for co-IP of type II phys, fully active phyA-m1 in line 135 and partially active phyA-m6 in lines 136 and 137. No evidence for co-precipitation of any of the type II phys with either the phyA-myc1 or phyA-myc6 proteins was obtained from extracts of dark-grown seedlings, where phyA is cytosolic and present as Pr. Supporting S4 Fig shows that the same result is obtained following immunoprecipitation of tagged phyA from seedlings grown for 24 h under continuous FR, light conditions that induce FR-HIR and where phyA is predicted to be 1–2% Pfr and primarily nuclear [16, 41]. Therefore, in contrast to the complex pattern of heterodimerization seen in type II phys [29], type I phyA in Arabidopsis in its Pr form appears to be a discrete population of homodimers. Moreover, no evidence for physical association of phyA Pr with type II phy homo- or heterodimers in a stable dimer-to-dimer higher-order structure was observed under the conditions tested.

**Fig 6:**
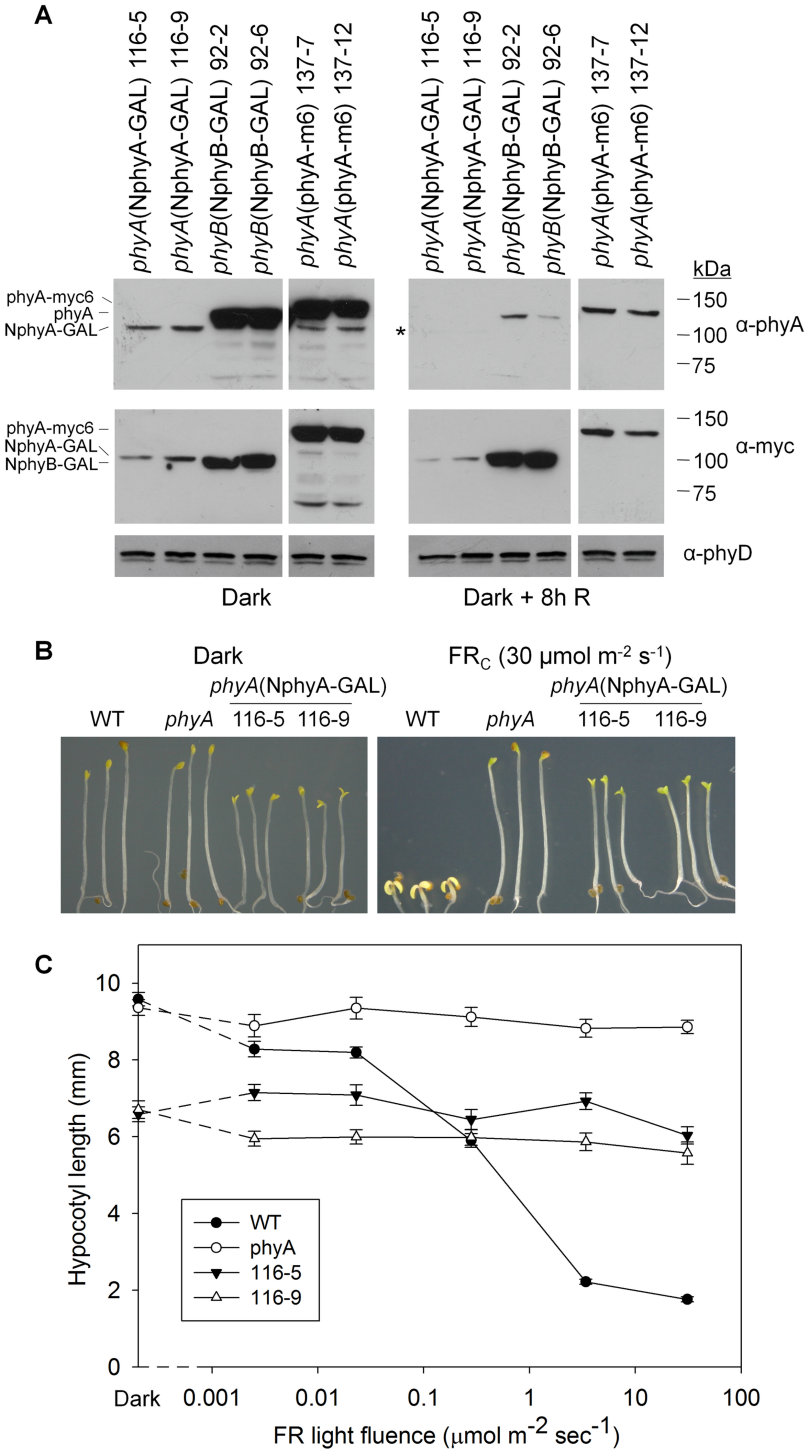
Levels of the NphyA-GAL-myc6-NLS protein in transgenic lines and the *cop* phenotype associated with its expression. (A) Immunoblot analysis of the levels of the NphyA-GAL and NphyB-GAL fusion proteins in dark-grown seedlings and in seedlings exposed to 8 h of R light. The NphyA-GAL protein is detected by both the anti-phyA and anti-c-Myc Abs in the #116 lines, and the NphyB-GAL protein is detected by the anti-c-Myc Ab in the #92 lines. Native phyA is detected by the anti-phyA Ab in the *phyB*(NphyB-GAL) #92 lines, and the phyA-myc6 protein is detected by both the anti-phyA and anti-c-Myc Abs in the *phyA*(phyA-m6) #137 lines. The signal for the NphyA-GAL protein in the #116 lines under 8h R was barely visible on the blot probed with the anti-phyA Ab on this exposure (*) but was detected on longer exposures. The anti-phyD immunoblot is a loading control. (B) Phenotypes of seedlings of the indicated genotypes grown in darkness or continuous FR. Seeds were stratified, induced to germinate with a pulse of R (30 μmol m^-2^ s^-1^), and incubated at 22°C for 4 days in darkness or for one day in the dark followed by 3 days under FR. (C) FR fluence response curve showing the lack of effect of continuous FR on the mild *cop* phenotype of NphyA-GAL #116 lines. Seedlings were grown for one day in the dark followed by 3 days under continuous FR of the indicated fluence and hypocotyls were measured (±SE; n = 20–30). In unpaired t-test analysis, all *p*-values are less than 0.0001 except when comparing wild-type to *phyA* or comparing the two transgenic lines in the dark, the wild-type to *phyA* under 0.0025 μmol m^-1^ s^-2^ FR, and the wild-type and the two transgenic lines at 0.28 μmol m^-1^ s^-2^ FR (*p* > 0.1).

Consistent with phyA functioning as a homodimer, previous studies have shown that fusion of detached phyA N-terminal PSD sequences (NphyA) to heterologous dimerization domains, a nuclear localization sequence (NLS), and YFP generates chimeric molecules that have varying light-regulated activities in transgenic plants. In such constructs, the oat NphyA(1–595), Arabidopsis NphyA(1–686), and Arabidopsis NphyA(1–406) PSD regions all fail to mediate the FR-HIR hypocotyl elongation response and have varying activities in the VLFR [27, 38, 42]. Surprisingly, some of these fusion gene lines show a *constitutively photomorphogenic* (*cop*) phenotype in the dark [27, 38], while others do not [42]. We have observed here that even relatively small modifications of the ends of the phyA apoprotein can affect its function so, to further the earlier studies, we analyzed the activity of an Arabidopsis NphyA(1–615) PSD sequence using a yeast domain-mediated homodimerization system [31]. Arabidopsis NphyA(1–615), which matches the amino acid sequence coordinates of the NphyB, NphyC, NphyD, and NphyE PSD regions described previously [30, 31], was fused to the yeast GAL4 homodimerization domain, a myc6 epitope tag, and the SV40 NLS and this coding sequence was driven by the CaMV 35S promoter in *phyA* mutant transgenic lines (Fig 2A). Fig 6A shows the levels of native phyA, the phyA-myc6 protein, the NphyA-GAL protein, and the NphyB-GAL protein in the dark and following 8 h of R. The chimeric NphyA-GAL protein is expressed at a low level compared to native phyA in the dark, and, unlike native phyA, is quite stable following transfer to R. The steady-state level of NphyA-GAL is also much lower than that of the NphyB-GAL protein [31], indicating that in both the presence and absence of light, these completely equivalent NphyA and NphyB fusion proteins have markedly different stabilities.

Fig 6B shows that the 35S:NphyA(1–615)-GAL transgene causes a significant *cop* phenotype in the dark, with shorter hypocotyls and more opened cotyledons compared to WT, and Fig 6C shows that it does not mediate a FR-HIR over the course of a FR fluence curve. The transgenic NphyA-GAL seedlings grown under high fluence FR are nearly identical to those grown in the dark apart from increased cotyledon opening and slight greening, which are also seen in the *phyA* parent (Fig 6B). No such *cop* phenotype was observed in any of the type II NphyB-GAL through NphyE-GAL transgenic lines [31] Fig 4A shows that, like the epitope-tagged full-length phyA apoproteins, NphyA(1–615)-GAL mediates a robust VLFR seedling elongation response under hourly pulses of FR. With respect to the *cop* phenotype illustrated in Fig 6B, the “Dark” panel shows seedlings that were induced to germinate with a treatment of 3 days at 4°C in the dark followed by 5 min of R, then incubation at 22°C in the dark. When this experiment was repeated but the 5 min R pulse was followed by a 5 min FR pulse given 3 h later, to convert the phyA-E Pfr that was formed by the germination-inducing R pulse to mostly Pr before the dark growth period, the small extent of cotyledon opening seen in both the WT and *phyA* mutant seedlings was reversed by about ten degrees to a completely closed state (S5 Fig). This ten-degree FR reversal was also seen in the *phyA*(NphyA-GAL) lines, but most of the constitutive cotyledon opening was not reversed, demonstrating that this characteristic of the *cop* phenotype relates to an activity of NphyA-GAL in darkness.

## Discussion

Physiological and genetic experiments show that most aspects of plant growth and development are responsive to the presence, intensity, duration, ratio, and/or periodicity of R and FR light via the activities of the phytochromes. Among the different forms of phytochrome encoded in the genomes of angiosperm plants, type I phyA is light-labile and performs unique functions in FR-HIR and VLFR [3, 18] while type II phytochromes, phyB-phyE in Arabidopsis, are light-stable and control plant responses to the ratio of R to FR, such as shade responses [1, 17]. Importantly, for a given light-influenced plant developmental transition or response in a given plant species, there are often significant influences of several of the phys, including cooperative, synergistic, or antagonistic effects. [25, 43–45]. Many of these interactive effects are likely mediated through signaling components and mechanisms that are shared among the phys and which can positively or negatively influence each other. In addition to this, as seen throughout biology, heterodimerization or heteromultimerization can impart higher-order combinatorial diversity of quaternary structure and function within a related group of receptors. The four Arabidopsis type II phys have been shown to assemble into a variety of heterodimeric forms, which may account for some of the cooperative or opposing regulatory effects [28, 29]. For example, whereas in monocot plants phyC alone plays the predominant role in photoperiodic effects on flowering [46], in Arabidopsis, function of both phyB and phyC are required for short day delay of flowering [25]. PhyC appears to be present primarily as a phyB/phyC heterodimer in Arabidopsis [29], suggesting that, in one major group of plants phyC functions as a homodimer, while in another group it functions as a heterodimer with other type II phys. The effects of R/FR are also often integrated with effects of other wavelengths, notably blue and UV light as sensed by cryptochromes, phototropins, and UV RESISTANCE LOCUS 8 (UVR8) receptors [17], and with other environmental cues such as temperature [47]. Within this context, we sought here to address the question of whether type I phyA physically interacts with the type II phys, via heterodimerization, through formation of dimer/dimer interactions, or as components of large signaling complexes.

The immunoprecipitation approach used to investigate possible interactions of type I and type II phys depended on development of fully active phyA fusion transgenes and this proved to be challenging, with many of the phyA fusion proteins tested showing reduced signaling function, notably in the FR-HIR response. The activities of a set of epitope-tagged full-length phyA apoproteins were monitored and it was observed that addition of the YFP coding sequence to the C-terminus or an 87-amino acid long myc6 tag sequence to the N- or C-terminus alters phyA activity to a small but significant degree. In contrast, no effect of a 12-amino acid long myc1 tag was detectable in the assays performed here. This demonstrates that even relatively minor modifications of phytochrome N- or C- termini can influence their activities and care should be taken in utilizing such fusion constructs in experiments.

In previous observations of heterodimerization of type II phyB-E, no co-precipitation of phyA with epitope-tagged phyB, phyC, or phyE was observed [28, 29]. This suggested that phyA does not physically interact with type II phys in a strong enough manner to be pulled down with them. Using immunoprecipitation of a completely active myc1-tagged phyA fusion protein from extracts of dark-grown or continuous FR-grown seedlings, the current experiments demonstrate that phyA in its Pr form homodimerizes but that, as previously seen, no co-precipitation of phyA with any of the phyB-E type II phys is detected. This indicates that both cytosolic and nuclear phyA Pr lacks stable interaction with phyB-E. Interestingly, Sanchez-Lamas et al [25] have used bimolecular fluorescence (BiFC) to show that phyA interacts with phyB, phyD, and phyE in the nuclei of tobacco cells that are transiently expressing fusions of these phys to the N- and C-termini of EYFP. Therefore, it is possible that type I/type II phy heterodimers or higher-order complexes form in the nucleus under light-grown conditions. Further immunoprecipitation experiments will need to be done to assess possible interaction of phyA Pfr, formed under red light before it is degraded, with the type II phys. Alternatively, it is known that relatively weak and/or transient protein interactions can be detected by BiFC, with half-lives too short to be isolated by a pull-down assay [48, 49]. If this is the case for the phys, the BiFC-detectable interactions [25] may represent novel phy type I and II functional interactions that relate to nuclear signaling pathways, receptor turnover, or photochemical stabilization.

Fusion of the 550–700 amino acid-long plant phy N-terminal photosensory domains (PSD) to heterologous protein sequences that mediate dimerization and constitutive nuclear localization generates chimeric receptors with varying degrees of regulatory activity in plants. This shows that the C-terminal halves of phys are not essential to many of the major events of phy signaling as long as the PSD is dimerized and present in the nucleus. This was first demonstrated for phyB and has since been shown for type II phyC-E [26, 30, 31]. Because in our current experiments phyA appears to be uniquely homodimeric, directed dimerization and nuclear targeting of the phyA PSD might be expected to reproduce the activity of full-length phyA to a high extent, but previous results with NphyA PSD fusions have been mixed. Fusion of the oat phyA N-terminal 1–595 amino acid PSD to the GFP, GUS, and SV40-NLS sequences generated a light-stable chimeric molecule, with nearly identical photochemistry to full-length phyA, which mediated the VLFR but not the FR-HIR in transgenic Arabidopsis [27]. This chimeric molecule also caused a mild *cop*-like phenotype in darkness. Fusion of a 1–686 amino acid Arabidopsis phyA PSD to the CPRF transcription factor dimerization domain, YFP, and the SV40-NLS produced a fusion protein that failed to mediate the FR-HIR or the nuclear VLFR, and did not cause a *cop* phenotype [42]. In the light, neither of these NphyA fusions was observed to associate with nuclear bodies, which are characteristic of full-length phyA-YFP localization following conversion to Pfr [36]. A much shorter 1–406 Arabidopsis phyA PSD, in the same protein fusion construct as the 1–686 PSD, was inactive in FR-HIR signaling but induced a strong *cop* phenotype, indicating that it partially relieved the repression of photomorphogenesis by the COP/DET/FUS and/or PIF systems, and formed nuclear bodies both in darkness and after a R pulse [38].

In order to assess the activity of a 35S:NphyA fusion protein using a PSD region equivalent to the previously described NphyB-E constructs [26, 30, 31], we analyzed an NphyA(1–615)-GAL-myc6-NLS fusion. This chimeric protein was stable in the light, active in VLFR, inactive in FR-HIR, and caused a distinct *cop* phenotype. The potential association of the NphyA(1–615) chimera with nuclear bodies could not be determined because no fluorescent protein sequence was included in the transgene. Overall, the biological activity of the 88 kD NphyA(1–615)-GAL-myc6-NLS construct was more similar to that of the much larger ~163 kD oat NphyA(1–595)-GFP–GUS–NLS protein [27] than to the ~110 kD NphyA(1–686)-YFP-DD-NLS fusion, which did not mediate VLFR or cause a *cop* phenotype [42]. It may be significant that all the NphyA fusions that cause a *cop* phenotype were driven by the CaMV 35S promoter, whereas the NphyA(1–686)-YFP-DD-NLS fusion was driven by the native *PHYA* promoter. Whether it is ectopic NphyA expression that causes the *cop* phenotype will need to be tested. It is, however, clear that the homodimerized phyA N-terminus in all of these constructs is very deficient in signaling in the FR-HIR, a classical phyA-mediated response. We conclude that the truncated length of the phyA sequence present in these chimeric molecules and the heterologous domains used to dimerize them strongly influences their molecular properties and activities, and that the active full-length homodimer state of phyA is not accurately simulated in these proteins.

## Supporting information

S1 Fig Coding sequences of the amino-termini or carboxy-termini of the prom*PHYA*-phyA transgene and the c-Myc epitope-tagged phyA transgenes. The c-Myc (myc) tags are shown as underlined and *PHYA* coding sequences are in bold.
(TIF)

S2 Fig Expression of epitope-tagged phyA fusion proteins under continuous FR light. WT, *phyA* mutant, and the indicated *phyA*(*PHYA*:phyA-tagged) transgenic lines were grown for one day in darkness and 4 days under FR (4 μmol m^-2^ s^-1^). Protein extracts of seedlings were fractionated on SDS gels, blotted, and probed with the indicated antibodies. *, the myc6-phyA protein is degraded to a lower molecular weight under FR.
(TIF)

S3 Fig Immunoblot analysis of the stability of epitope-tagged phyA fusion gene products following transfer from dark to red light. Seedlings were grown for 5 days in the dark, then transferred to continuous R (30 μmol m^-2^ s^-1^) for the indicated times. Protein extracts were prepared, fractionated on 7% SDS gels, blotted, and probed with the anti-phyA antibody. Four sample replicate immunoblots for the WT extracts, two sample replicate blots for the 135, 137, and 103 epitope-tagged line extracts, and one immunoblot for the 136 extracts were performed. The blots were scanned and densitometry was performed using ImageJ software. Representative immunoblots are shown. One anti-phyD control immunoblot was performed for each set of extracts. Band intensity values for each point on the curves are the averages of the relative densitometry readings for the replicate blots for that data point, with the dark reading set as 1, divided by the relative densitometry reading for the phyD control blot for that data point (±SE).
(TIF)

S4 Fig Immunoblot analysis of FR-grown seedling extracts and anti-c-Myc antibody immunoprecipitates from WT and lines expressing the indicated transgenes. Seedlings were grown for 4 days in the dark followed by 24 h in continuous FR (31 μmol m^-2^ s^-1^). The WT(phyA-m6) extract contains both native phyA and the higher molecular weight myc6-tagged phyA.
(TIF)

S5 Fig Cotyledon angles of the seedlings (±SE; n = 20–30) shown in Fig 6B and 6C. Seeds were stratified, induced to germinate with a pulse of R (30 μmol m^-2^ s^-1^), and incubated at 22°C for 4 days in darkness (A) or for one day in the dark followed by 3 days under FR (B). A parallel experiment to that done in (A) was performed in which the R pulse used to induce germination was followed by 3 h in the dark and a pulse of FR (31 μmolm^-2^s^-1^) prior to incubation for 4 days in darkness. Cotyledon angles from these seedlings (C) show an approximately 10 degree reversal of cotyledon opening compared to seedlings in (A) but the transgenic 35S:NphyA-GAL seedlings continue to show a constitutive increase in cotyledon angle. In unpaired t-test analysis, all *p*-values are less than 0.05 except when comparing the transgenic 116 lines under conditions (A) and (C), where *p* > 0.1.
(TIF)
