# Supplementary figures and images for: Biological activity and dimerization state of modified phytochrome A proteins
Source: PLoS One. 2017 Oct 19;12(10):e0186468. doi: 10.1371/journal.pone.0186468 (PMC5648194; doi:10.1371/journal.pone.0186468)

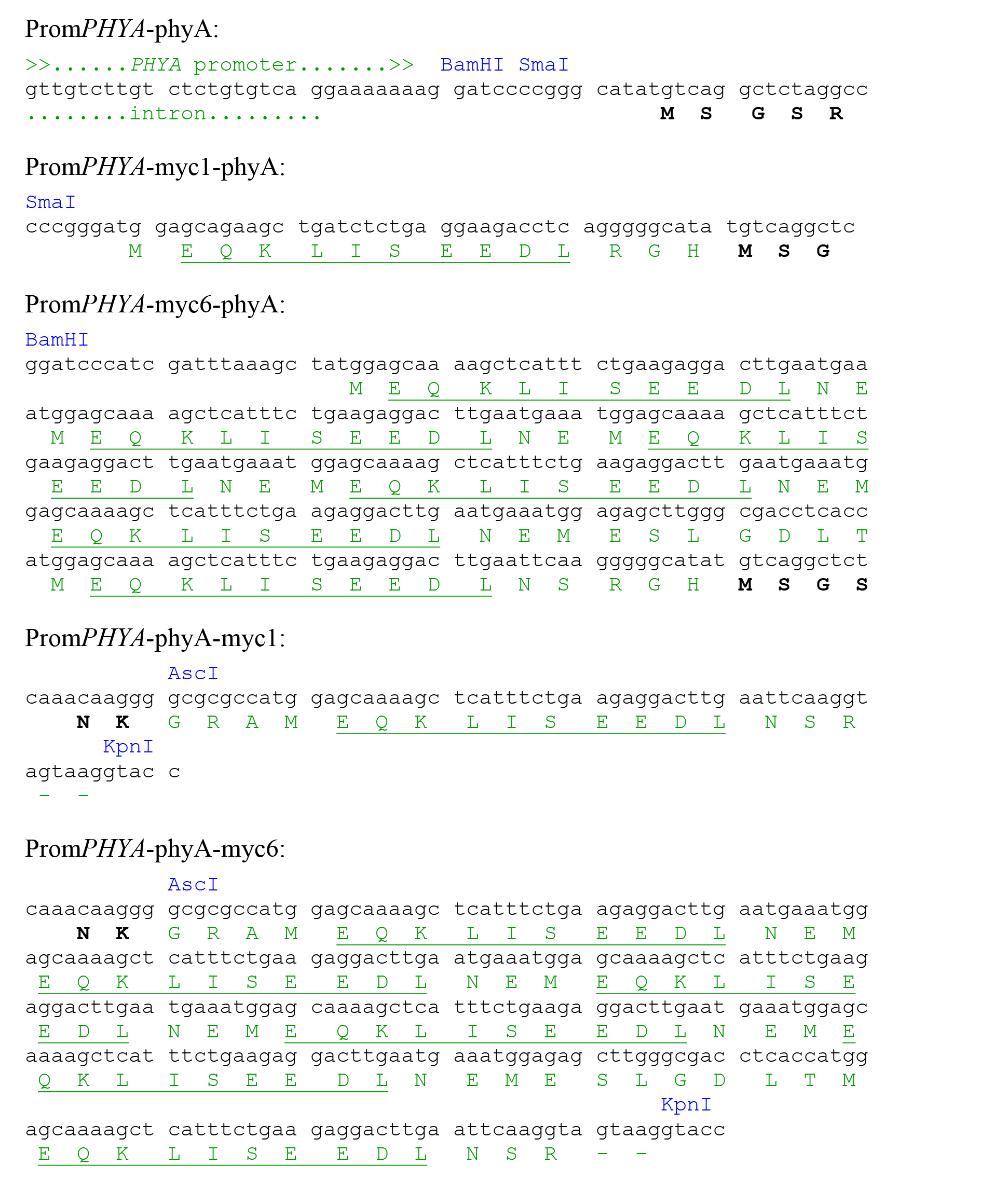

Supplement: S1 Fig — The c-Myc (myc) tags are shown as underlined and PHYA coding sequences are in bold. (TIF) [file pone.0186468.s001.tif]

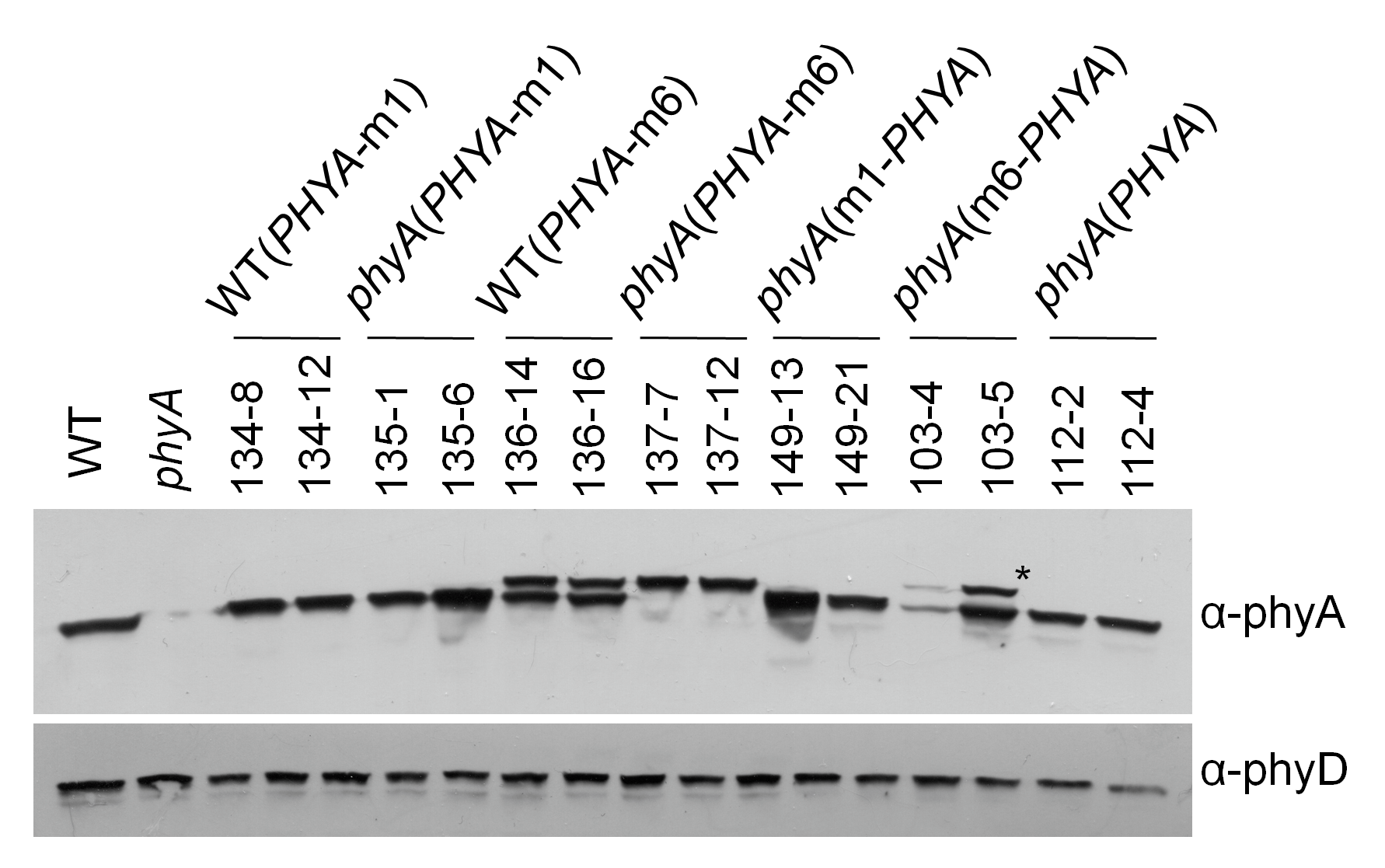

Supplement: S2 Fig — WT, phyA mutant, and the indicated phyA(PHYA:phyA-tagged) transgenic lines were grown for one day in darkness and 4 days under FR (4 μmol m-2 s-1). Protein extracts of seedlings were fractionated on SDS gels, blotted, and probed with the indicated antibodies. *, the myc6-phyA protein is degraded to a lower molecular weight under FR. (TIF) [file pone.0186468.s002.tif]

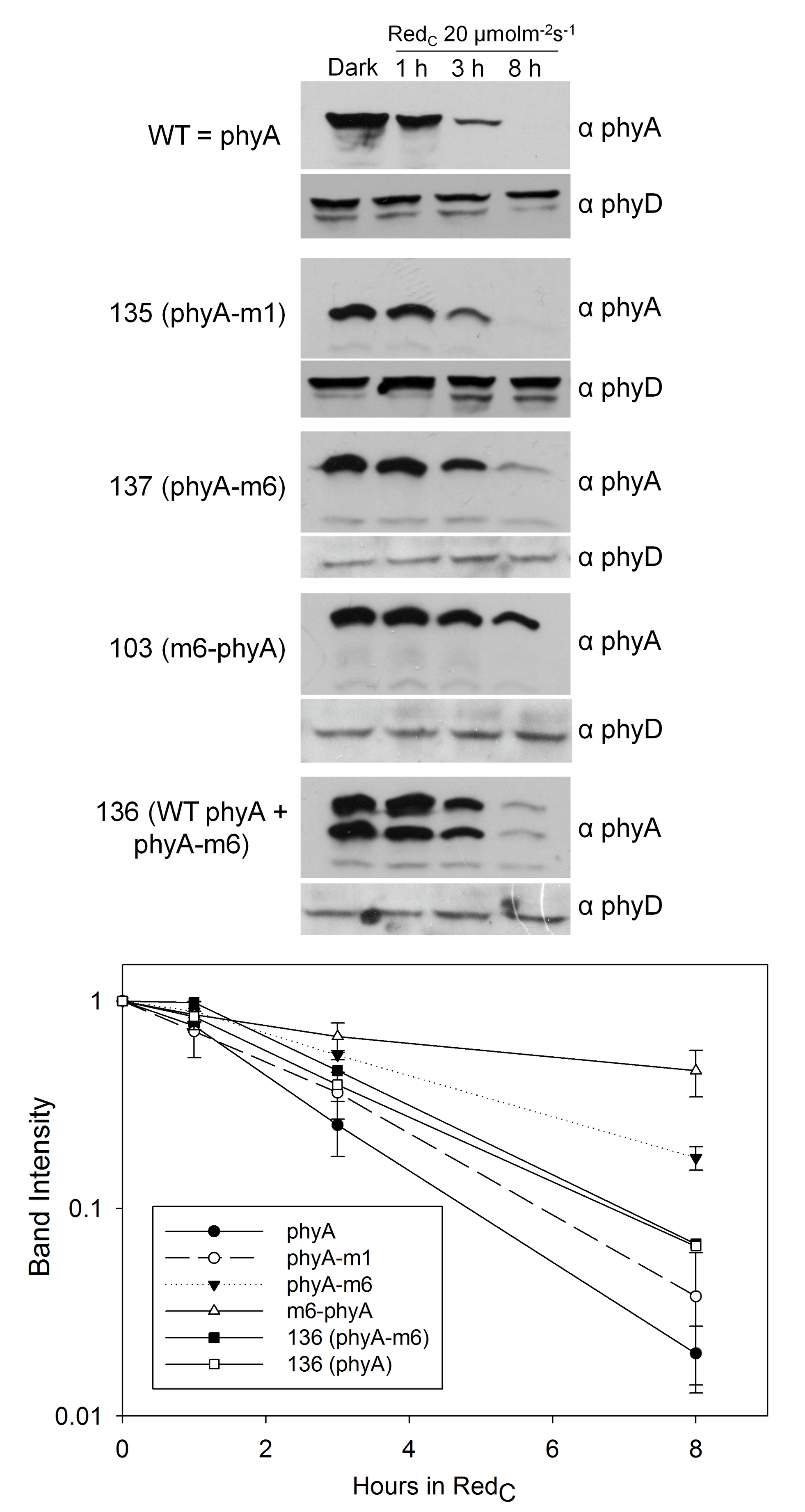

Supplement: S3 Fig — Seedlings were grown for 5 days in the dark, then transferred to continuous R (30 μmol m-2 s-1) for the indicated times. Protein extracts were prepared, fractionated on 7% SDS gels, blotted, and probed with the anti-phyA antibody. Four sample replicate immunoblots for the WT extracts, two sample replicate blots for the 135, 137, and 103 epitope-tagged line extracts, and one immunoblot for the 136 extracts were performed. The blots were scanned and densitometry was performed using ImageJ software. Representative immunoblots are shown. One anti-phyD control immunoblot was performed for each set of extracts. Band intensity values for each point on the curves are the averages of the relative densitometry readings for the replicate blots for that data point, with the dark reading set as 1, divided by the relative densitometry reading for the phyD control blot for that data point (±SE). (TIF) [file pone.0186468.s003.tif]

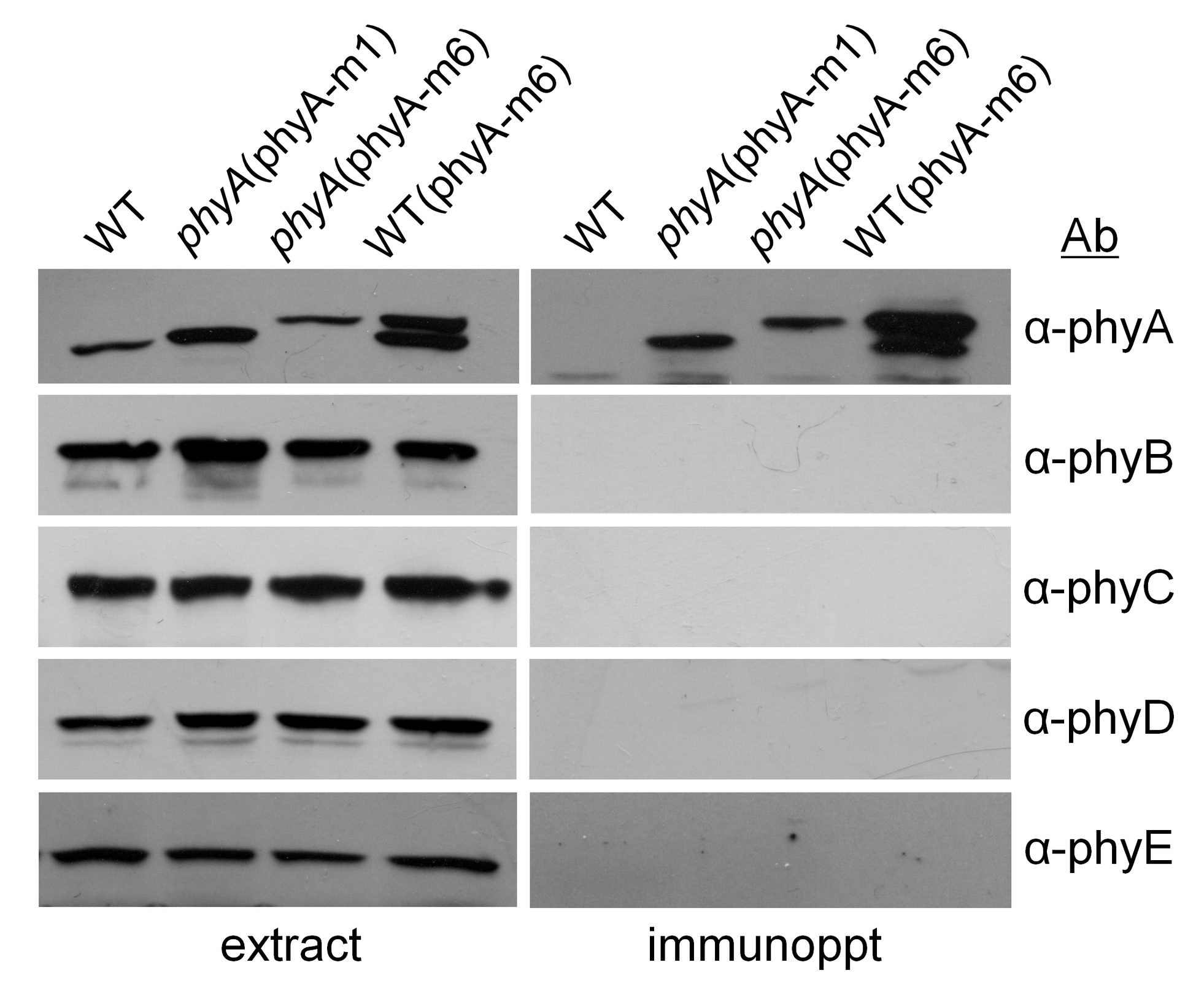

Supplement: S4 Fig — Seedlings were grown for 4 days in the dark followed by 24 h in continuous FR (31 μmol m-2 s-1). The WT(phyA-m6) extract contains both native phyA and the higher molecular weight myc6-tagged phyA. (TIF) [file pone.0186468.s004.tif]

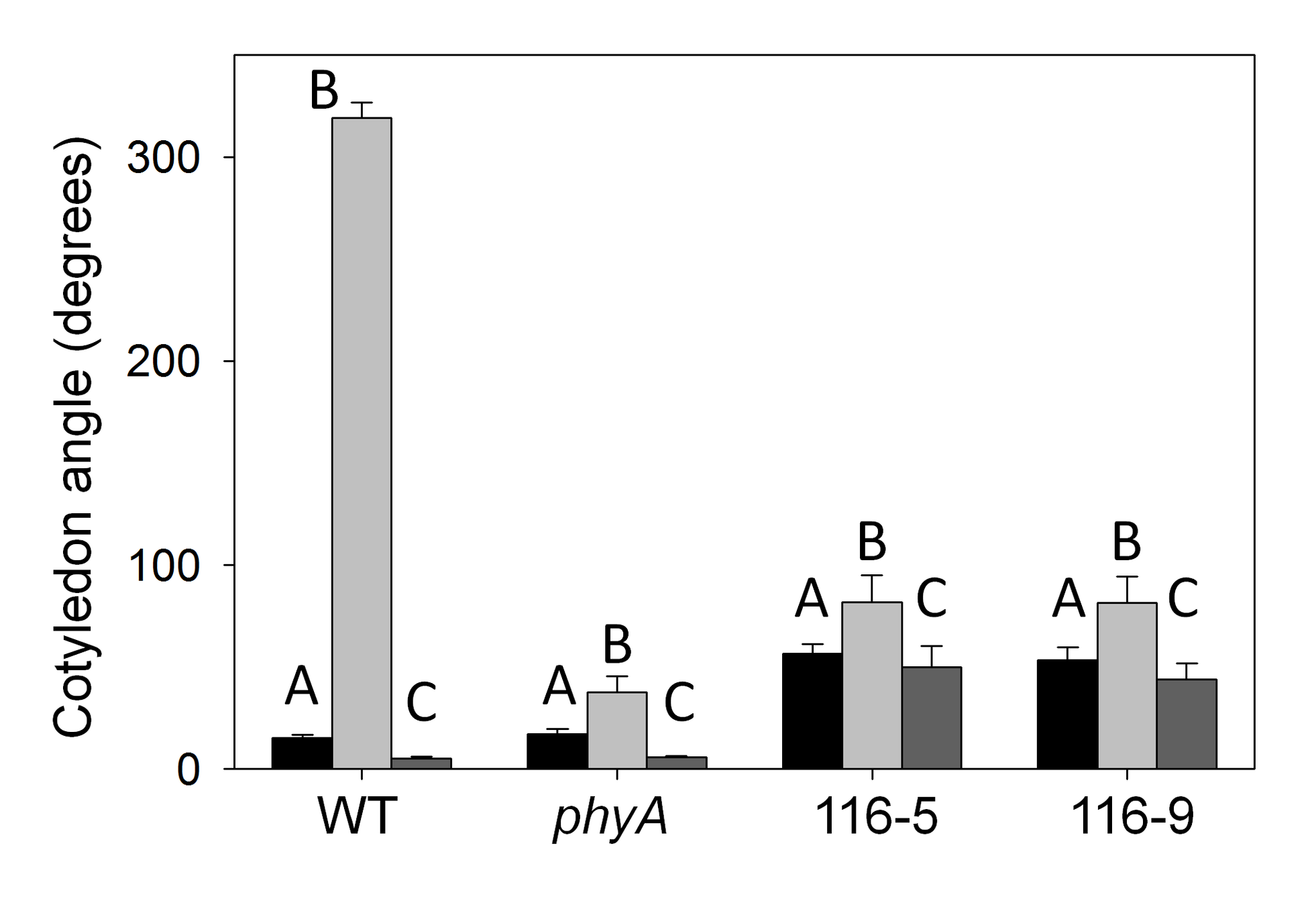

Supplement: S5 Fig — Seeds were stratified, induced to germinate with a pulse of R (30 μmol m-2 s-1), and incubated at 22°C for 4 days in darkness (A) or for one day in the dark followed by 3 days under FR (B). A parallel experiment to that done in (A) was performed in which the R pulse used to induce germination was followed by 3 h in the dark and a pulse of FR (31 μmolm-2s-1) prior to incubation for 4 days in darkness. Cotyledon angles from these seedlings (C) show an approximately 10 degree reversal of cotyledon opening compared to seedlings in (A) but the transgenic 35S:NphyA-GAL seedlings continue to show a constitutive increase in cotyledon angle. In unpaired t-test analysis, all p-values are less than 0.05 except when comparing the transgenic 116 lines under conditions (A) and (C), where p > 0.1. (TIF) [file pone.0186468.s005.tif]
